# Supplementary material for: Decoding the content of working memory in school-aged children
Source: bioRxiv. 2023 Feb 12:2023.02.10.527990. Preprint. [Version 1] doi: 10.1101/2023.02.10.527990 (PMC9934641; doi:10.1101/2023.02.10.527990)
Supplement: Supplementary file 1 [file media-1.docx]

**Decoding the content of working memory in school-aged children**

**Supplementary Materials**

**Supplemental EEG classification results: time-wise decoding using 20ms time-bin averages against theoretical chance**

A classifier was trained and tested on data from smaller 20ms time bins steps over a trial (without the Baseline period, i.e. 0 to 3000ms), where EEG voltage data were averaged over each time bin. The average decoding accuracy over the duration of a trial was 48.7% (median = 49.2%, *SD* = 9%), which was significantly above chance (33%, *p* *=* 5.82e^-11^; Supplementary Figure 1A). Consistent with the 50ms time bin results, we found significant above-chance decoding throughout nearly all of the Sensory period, and until almost 2000ms into the Delay period, but no above-chance decoding during the Baseline period. All of these results were significant according to a Bonferroni-corrected paired *t*-test with 1000 permutations (corrected *p*-value was 3.05e^-04^). 

	Next, a temporal generalization classification examined the unfolding of information category representations across time. Consistent with the 50ms time bin results, we found that representations that were detected during the early Sensory period could be detected over the rest of the Sensory period, and for a portion of the Delay period. This is reflected in three patterns per Supplementary Figure 1B: 1) the high accuracy levels on the main diagonal on the graph, 2) data trained on Delay period representations showing higher accuracy when tested on early Sensory period data, and 3) data trained on early Sensory period representations showing somewhat high accuracy when tested on Delay period data.

The combined time-wise results using 20ms time bins corroborate the results of the 50ms time bin analysis. First, the results show that observed and maintained representational content can be decoded from children’s EEG. Second, they show that the same representational structures persist even in the absence of stimuli, during their maintenance in WM. 

**Supplementary Figure 1.** A) Timepoint-by-timepoint classification results using 20ms time bins. The average across participants is depicted in a blue line (time points that were significant after Bonferroni correction are represented by a thicker blue line). The standard deviation is depicted in the shaded blue area. Theoretical chance (33%) is depicted in a black dashed horizontal line, and the onsets of the Sensory (0ms) and Delay periods (1000ms) relative to stimulus onset are depicted in dashed green and purple vertical lines, respectively. B) Temporal generalization of classification performance using 20ms time bins. Average classification values are overlaid by a significance mask: dark blue fields indicate the time points at which classification did not survive the cluster-level correction for multiple comparisons, while all other colors correspond to above-chance classification generalization.

**Supplemental EEG classification results: time-wise decoding against empirical chance**

We conducted a time-wise decoding analysis comparing decoding accuracy against chance levels determined by permutated data (as opposed to the theoretical chance level of 33% as used in the main analyses). This analysis revealed similar results as the main time-wise analysis, in that the average decoding accuracy was mostly above chance over the course of a trial (see Supplementary Figure 2A for 50ms time-bin averages, and Supplementary Figure 2B for 20ms time-bin averages). Namely, while no significant above-chance decoding was observed during the Baseline period, there was significant above-chance decoding throughout nearly all of the Sensory period, and until around the first 1500ms into the Delay period. These results further support that multivariate representations of information categories are distinguishable during maintenance.  

**Supplementary Figure 2.**  Moment-by-moment classification results comparing original EEG voltage data to shuffled data. For the original data, average classification results are depicted in a blue line, and the standard deviation is depicted in the shaded blue area. For the ‘shuffled’ data, average classification results are depicted in a black line, and the standard deviation is depicted in the shaded gray area. Theoretical chance (33%) is shown with a turquoise dashed line, which is overlaid by the black average accuracy line of the shuffled data, showing that the latter is at theoretical chance. The onsets of the Sensory (0ms) and Delay periods (1000ms) relative to stimulus onset are depicted in dashed green and purple vertical lines, respectively. Panel A) shows the results of the 50ms time bin analysis, while panel B) the results of the 20ms time-bin analysis.

**Supplemental exploratory reliability and validity results: Split-half analyses using 20ms time-bin averages**

As in the 50ms time bin analyses, datasets were split into early (‘blocks’ 1 – 4 in the testing session) and late (‘blocks’ 5 – 8) halves, on which the same classification schemes as above were applied separately. Even when training/testing only within the early half (Supplementary Figure 3A), or only within the late half (Supplementary Figure 3B), classification was robust during almost the whole Sensory period, and within the first 500ms of the Delay period, with no above-chance decoding during the Baseline period. Thus, our method can reliably detect representations in a stable manner across the testing session, even when provided access to only half of the data.

To investigate the stability of representational structures from the beginning to the end of the session, we conducted a cross-decoding analysis of the early versus the late halves. With both 50ms and 20ms time bins, we observed reliable cross-decoding during the entire Sensory period and the first 500ms of the Delay period (Supplementary Figure 3C). This suggests that representational structures detected in the early half of the testing session generalize for a portion of the late half of the testing session. 

**Supplementary Figure 3.** Classification performance on EEG data separated into early (blocks 1-4) and late (blocks 5-8) halves. A) shows early half classification results, B) shows late half classification results, and C) shows cross-decoding results between the early and late halves. Across all panels, average accuracy is depicted in a blue line (time points that were significant after Bonferroni correction are represented by a thicker blue line). The standard deviation is depicted in the shaded blue area. Theoretical chance (33%) is depicted in a black dashed horizontal line, and the onsets of the Sensory (0ms) and Delay periods (1000ms) relative to stimulus onset are depicted in dashed green and purple vertical lines, respectively.
